# Case Report: Therapeutic potential of traditional Korean herbal medicine Jeopgol-tang in bone regeneration: a case series of delayed union over 5 months

**DOI:** 10.3389/fendo.2025.1595784

**Published:** 2025-06-30

**Authors:** Eunbyul Cho, Seri Lee, Youngjin Choi, Changsop Yang, Hyeonjun Woo

**Affiliations:** ^1^ Korean Medicine (KM) Science Research Division, Korea Institute of Oriental Medicine, Daejeon, Republic of Korea; ^2^ Department of Diagnostics, College of Korean Medicine, Wonkwang University, Iksan, Republic of Korea; ^3^ College of Korean Medicine, Dongguk University, Goyang, Republic of Korea; ^4^ Kyunghee Dabok Korean Medicine Clinic, Seoul, Republic of Korea; ^5^ Department of Korean Medicine Rehabilitation, College of Korean Medicine, Semyung University, Jecheon, Republic of Korea

**Keywords:** fracture, delayed union, herbal medicine, Jeopgol-tang, case report

## Abstract

The delayed union of fractures poses considerable challenges in orthopedic treatment, affecting patients’ quality of life and healthcare outcomes. Although various treatment options are available, their efficacy may be limited in patients with prolonged delayed union. This study investigated the therapeutic potential of Jeopgol-tang (JGT), a traditional herbal decoction, for treating cases of delayed union persisting for more than 5 months. Three patients with different fracture sites (pubic ramus, multiple metatarsals, and femoral neck) who exhibited insufficient healing after conventional treatment were administered JGT (100 mL per pack, twice daily) for 2–6 months depending on individual healing progress and fracture complexity. Radiological assessments demonstrated progressive improvement in all cases. Notably, one patient avoided reoperation after exhibiting initial signs of bone healing within 1 month of treatment, whereas all patients achieved complete union and reported improved mobility and reduced pain. These findings suggest that JGT could be an effective complementary treatment option for delayed union, particularly in cases in which conventional treatments have yielded limited success. Nevertheless, larger prospective clinical trials and mechanistic studies are required to validate these results and to explore the therapeutic mechanisms of JGT.

## 1 Introduction

Bone-fracture healing is a complex biological process involving a series of coordinated molecular and cellular events (1). Although a direct healing pattern can occur when the fracture is reduced and remains nearly completely stable, most cases follow an indirect healing pattern consisting of both endochondral and intramembranous bone healing. This process typically progresses through four distinct phases that include inflammation (hematoma formation), granulation tissue formation, bony callus formation, and bone remodeling (2–4).

When this intricate healing process is disrupted by inadequate immobilization, compromised blood supply, infection, or nutritional deficiency, delayed union or nonunion may occur (4). Delayed union, typically diagnosed when a fracture exhibits no radiological signs of healing between 3- and 6- months post-injury, occurs in 10–15% of surgically managed fracture cases and poses considerable challenges for both patients and healthcare providers (5). Current treatment approaches, including surgical intervention, biophysical stimulation, and local biological enhancement, often result in limited efficacy and may be associated with complications (6–8).

Herbal medicines have been used for fracture healing and treatment of symptoms related to fractures across East Asia for thousands of years (9). Large population cohort studies have revealed a significant association between the use of herbal medicine and a reduced risk of osteoporotic fractures as well as a lower risk of overall mortality, readmission, and reoperation (10, 11). Recent studies have elucidated the mechanisms underlying the effectiveness of these traditional approaches. Herbal medicines can enhance bone formation through multiple mechanisms (12) and pathways, including regulation of osteogenic differentiation (13), modulation of inflammatory responses (14), and promotion of angiogenesis (15, 16).

Jeopgol-tang (JGT), a traditional herbal decoction used for fracture treatment, prepared through non-pressure hot water extraction of various herbs including *Angelicae Gigantis Radix*, *Cnidii Rhizoma*, and *Achyranthis Radix*. This herbal formula has exhibited promising results in the context of bone healing and regeneration. In Sprague–Dawley rats, JGT administration resulted in significantly enhanced callus growth within 2 weeks and increased callus length after 4 weeks compared to that of controls (17). Additionally, safety assessments confirmed that JGT does not induce acute toxicity or adverse effects (18). High-performance liquid chromatography identified nodakenin, ferulic acid, and dipsanoside A as the major compounds in JGT (18). Clinical studies have demonstrated that JGT improves bone density (19) and is effective for delayed union as early as 3 months after the fracture (20).

The present study aimed to investigate the therapeutic potential of JGT in treating cases of delayed union that persisted for more than 5 months. Herein, we present three cases in which conventional treatments failed to achieve adequate healing, focusing on the clinical outcomes and radiological evidence of bone regeneration following JGT administration.

## 2 Case presentation

### 2.1 Case 1

**Figure 1:**
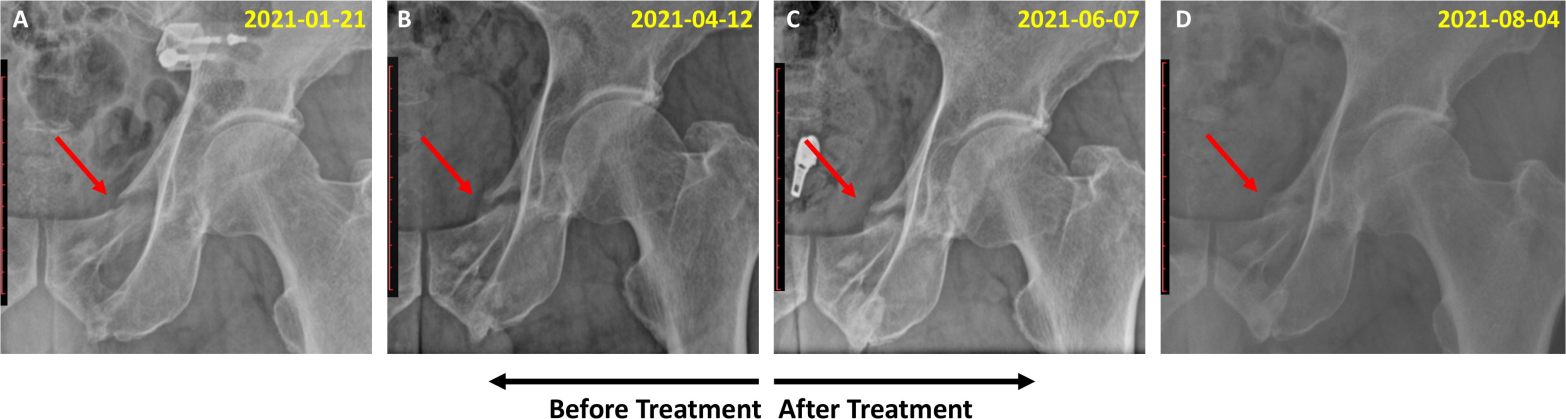
Radiographic images of the pelvis of Case 1, a 65-year-old man with a pubic ramus fracture. **(A)** Fracture condition of the pubic ramus at 3 months after the traffic accident (January 21, 2021); **(B)** Callus formation status 6 months after the fracture (April 12, 2021); **(C)** Soft callus formation 2 months after JGT administration (June 7, 2021); **(D)** Hard callus formation (August 4, 2021).

A 65-year-old man was diagnosed with a pubic ramus fracture based on radiographic images after a traffic accident on October 28, 2020. The patient experienced no underlying medical conditions. Owing to the mild displacement of the fracture, conservative treatment was performed at the initial hospital, including hospitalization for three weeks with acupuncture, herbal medicine for blood stasis, anti-inflammatory analgesics, and use of an abdominal binder across the waist and pelvis for stability support. However, callus formation was not complete until 6 months after the fracture. Accordingly, the patient visited our Korean Medicine clinic on April 21, 2021, and underwent JGT treatment for 2 months. On May 21, 2021, the patient received an injection of teriparatide (Forsteo) administered 11 times over 5 months to increase bone density. Radiography revealed callus growth, blurred fracture lines, and clear evidence of continuous callus formation (**Figure 1**). The improvement in fracture healing was more apparent after JGT treatment (**Figures 1C, D**) compared with the pretreatment state (**Figure 1B**). In November 2021, the patient was determined to have achieved complete recovery.

### 2.2 Case 2

**Figure 2:**
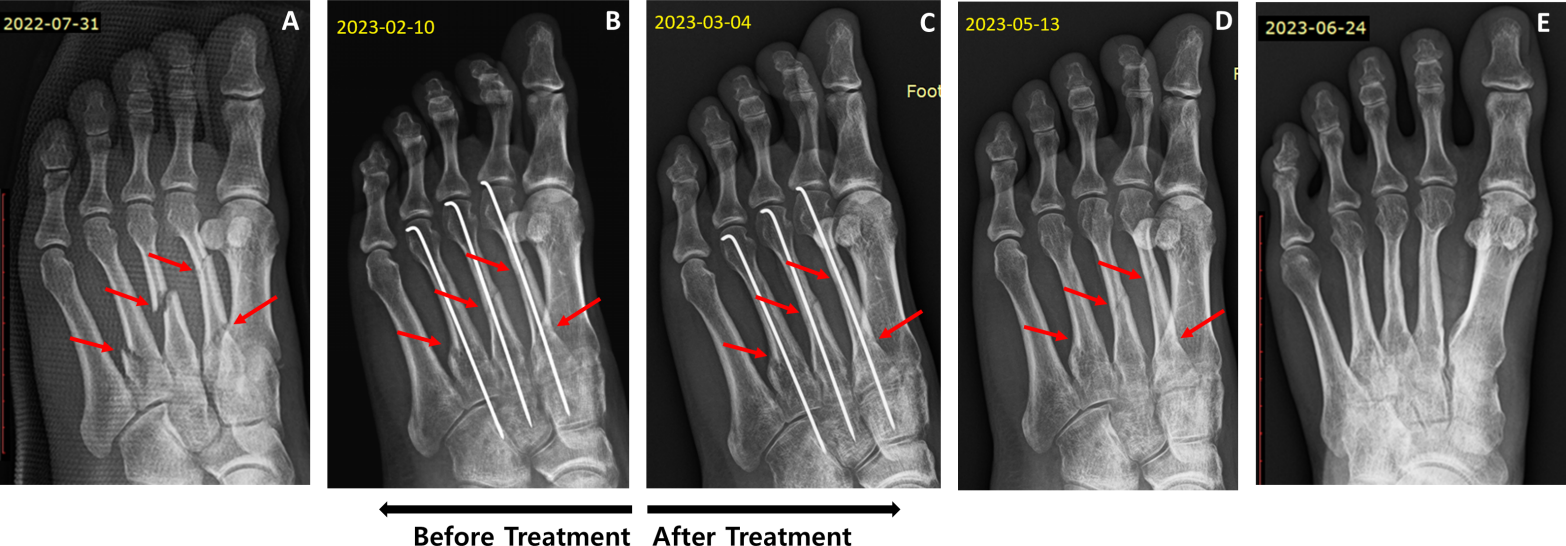
Radiographic images of the left foot of Case 2, a 30-year-old woman with multiple metatarsal fractures in the left foot. **(A)** Fracture condition before surgery (July 31, 2022); **(B)** Fracture status 6 months after surgery (February 10, 2023); **(C)** Fracture condition 1 month after JGT administration (March 4, 2023); **(D)** Fracture condition 3 months after JGT administration (May 13, 2023); **(E)** Bridging callus density similar to the bony cortex (June 24, 2023).

On July 30, 2022, a 30-year-old woman was diagnosed with fractures of the left second, third, and fourth metatarsals based on radiographic findings after falling while climbing (**Figure 2A**). The patient underwent internal fixation surgery the next day, was prescribed conventional medications, and used crutches for four months. However, callus formation was not identified until 6 months after surgery. Owing to incomplete bone union, the patient visited our clinic on February 11, 2023, and began JGT treatment. The patient underwent pin removal surgery on April 28, 2023, as the fracture had united, and underwent JGT for an additional 4 weeks from May 1, 2023. Before starting the herbal treatment, callus formation was insufficient (**Figure 2B**). Three months after JGT administration, radiographic imaging demonstrated evidence of union, characterized by bridging callus density similar to that of the bony cortex and a blurred fracture line (**Figure 2E**).

### 2.3 Case 3

**Figure 3:**
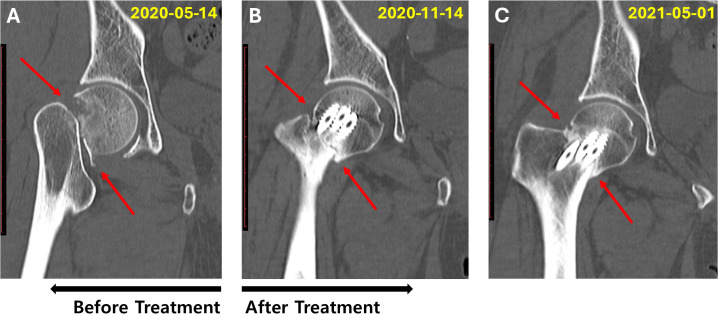
Computed tomography images of the right hip joint of Case 3, a 58-year-old woman with a femoral neck fracture. **(A)** Frontal planes of the right hip joint, showing the fractured femoral neck immediately after the accident and before surgery (May 14, 2020); **(B)** Frontal planes of the right hip joint 6 months after the fracture and 1 month after the diagnosis of delayed union (1-month follow-up after JGT administration) (November 14, 2020); **(C)** Frontal planes showing distinct changes in the right hip joint, demonstrating femoral neck fracture healing (May 1, 2021).

**Figure 4:**
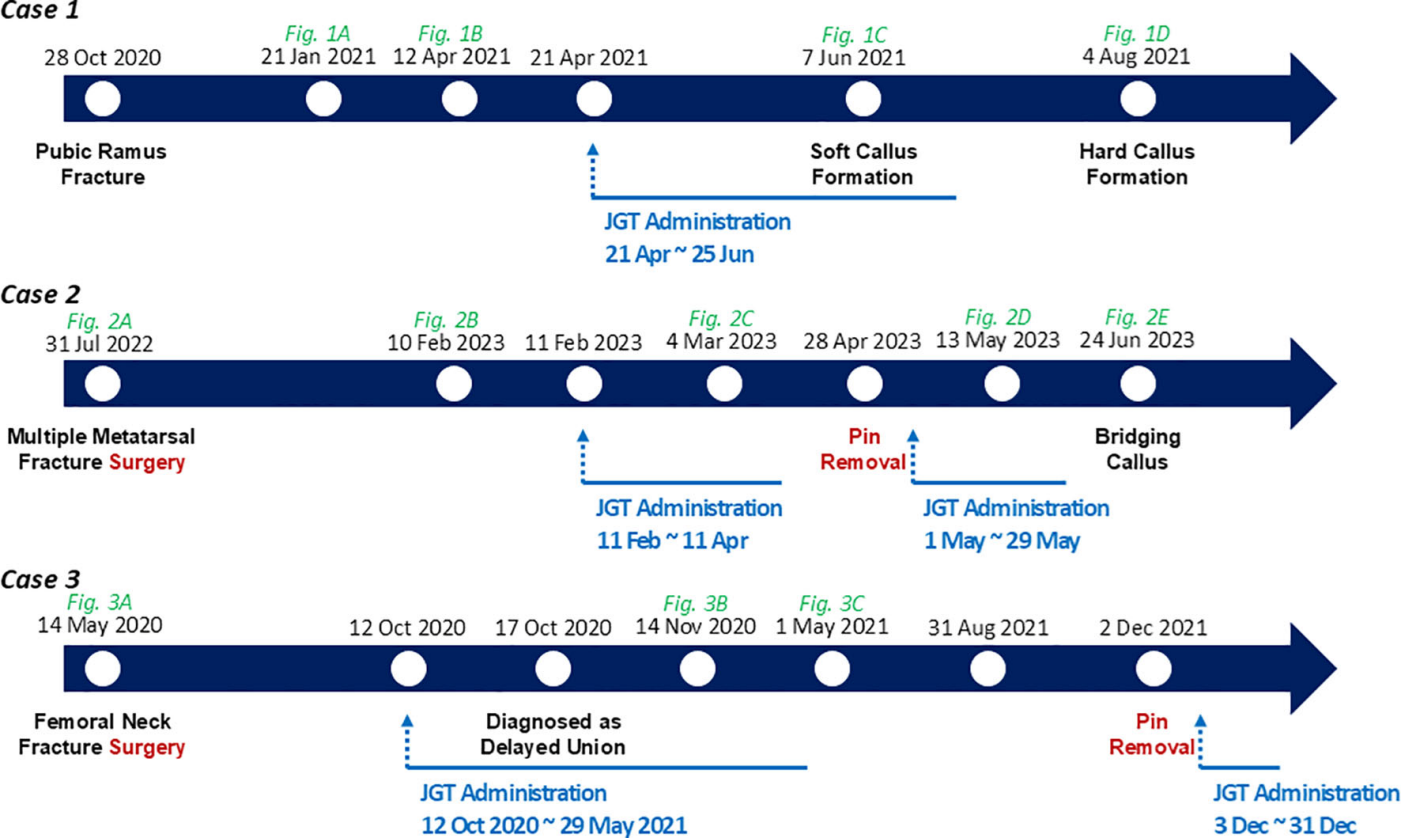
Timeline of Cases 1–3 treated with the herbal decoction Jeopgol-tang (JGT).

In May 2020, a 58-year-old woman was diagnosed with a fracture of the right femoral neck based on radiographic and computed tomography (CT) findings (**Figure 3A**). The fracture occurred when the patient fell while walking downstairs. The patient underwent internal fixation surgery in May 2020, and crutches were used daily. She also received osteoporosis injections and anti-inflammatory analgesics for 5 months. However, the attending physician diagnosed delayed union, as no callus formation or fracture improvement was observed on radiographic or CT images. The patient visited our clinic on October 12, 2020, for herbal medicine treatment to avoid reoperation and decided to wait another month before reconsidering surgery. On November 14, 2020, after 1 month of JGT administration, CT imaging indicated signs of improvement (**Figure 3B**), and she continued without reoperation. The patient was able to walk without crutches 4 months after the JGT treatment. On May 1, 2021, seven months after JGT administration, CT imaging revealed substantial fracture healing characterized by bridging callus formation across the fracture gap and restoration of cortical continuity (**Figure 3C**). She underwent pin removal surgery on December 2, 2021. The timelines for the three cases are presented in **Figure 4**.

### 2.4 Diagnostic assessment

The outcome of the fracture healing progression was assessed by a radiologist based on anteroposterior and lateral radiographs or CT images. No specific diagnostic challenges were observed.

### 2.5 Therapeutic intervention

All patients were administered two packs of the JGT decoction (100 mL per pack) daily. **Supplementary Table 1** presents the herbal composition of the JGT prescribed to each patient. In Case 2, *Zingiberis Rhizoma* and *Zizyphi Fructus* were added to aid digestion and address cold extremities. In Case 3, the dosage of *Citri Unshius Pericarpium* was increased from 8 to 16 g in Period 2 due to the patient’s complaints of difficulty sleeping. The same herbal prescription was maintained on December 14, 2020, owing to the improvement in sleep after increasing *Citri Unshius Pericarpium*. For depression (Case 3), *Perillae Folium* and *Coptidis Rhizoma* were included in Period 3. After pin removal (Case 3), herbal medicine was prescribed during Period 4 to address the deficiency. The patient maintained consistent adherence to the prescribed treatment, took JGT as directed, and attended all scheduled follow-up visits for continued prescription. None of the three patients experienced adverse events while taking herbal medicine. No other treatments such as physical therapy, acupuncture, or dietary strategies were provided during the JGT treatment at the clinic.

### 2.6 Patient perspectives

Case 1: “After I started taking JGT, I felt that the reduction of pain was happening faster. Also, I felt more comfortable walking.”

Case 2: “I did not feel uncomfortable when I started taking JGT. A revision operation was considered because callus formation was not detected six months after surgery. Fortunately, fracture union and pin removal were possible after JGT.”

Case 3: “There was no improvement or callus formation at the fracture site during internal fixation until 5 months after surgery. After 4 weeks of JGT treatment, minimal bone union was observed. I felt that my pain had reduced and walking had improved. Complete fracture healing was achieved without reoperation, and internal fixation was removed.”

## 3 Discussion

Fracture healing is a challenging and crucial process for both patients and clinicians (4). Fracture nonunion or delayed union directly affects the patients’ quality of life and their physical and mental health (8). As delayed union leads to a higher probability of nonunion, early and aggressive treatment is essential. This case report demonstrates the clinical effectiveness of JGT, a traditional herbal formulation, for treating delayed union lasting more than 5 months.

Experimental studies have been conducted on JGT extract, which consists of eight herbs at specific dosages, to evaluate its efficacy and safety (17, 18). Nodakenin, a marker compound of JGT, promotes osteoblast differentiation through activation of the PI3K/Akt/mTOR signaling pathway while simultaneously inhibiting osteoclastogenesis via suppression of c-Src/TRAF6/NF-κB pathways, demonstrating dual mechanisms crucial for bone remodeling and repair (21). Additionally, nodakenin has been demonstrated to exert anti-osteoporotic effects through comprehensive modulation of the gut-bone axis, including restoration of the Firmicutes/Bacteroidetes ratio, enhancement of beneficial bacterial strains such as Muribaculaceae and Allobaculum, promotion of calcitriol production and subsequent VDR activation, and improvement of intestinal barrier integrity through upregulation of tight junction proteins Occludin and ZO-1 while reducing pro-inflammatory cytokines IL-1β and TNF-α (22).

Ferulic acid, one of the major compounds in JGT, has been demonstrated to enhance vascular endothelial function by increasing nitric oxide bioavailability and promoting angiogenesis through the upregulation of vascular endothelial growth factor, platelet-derived growth factor, and hypoxia-inducible factor-1α expression (23). These vascular effects may contribute to bone healing by improving the blood supply and creating a favorable microenvironment for bone regeneration, particularly in cases of delayed union, where compromised vascularity often impedes the healing process. Furthermore, during fracture healing, key signaling molecules including TGF-β, IL-1, IL-6, TNF-α, and VEGF are released to orchestrate bone repair and vascular proliferation (4). Given that ferulic acid demonstrates pharmacological effects including TGF-β/Smad pathway modulation, regulation of inflammatory cytokines through NF-κB and p38 MAPK pathways, and enhancement of angiogenic factors (23), it may potentially influence bone union processes by optimizing the molecular environment beyond its direct vascular effects.

Thus, JGT may serve as an alternative treatment option for patients who are unsuitable for surgical intervention. In Case 1, a patient with a pubic ramus fracture, for whom the surgical approach was challenging, exhibited soft callus formation and eventually achieved complete union with JGT treatment. Furthermore, in Case 3, where reoperation was considered because of the lack of bone healing despite conventional treatments, including analgesics and osteoporosis injections, signs of fracture recovery were observed after 1 month of JGT administration, allowing the patient to avoid additional surgery. All patients reported high satisfaction with the treatment outcomes, as complete bone union was achieved after JGT treatment in cases where delayed union persisted for > 5 months. These findings suggest that JGT administration after acute fracture management may accelerate healing.

This study had several limitations. First, the results of these three cases cannot be generalized to a broader population. Large-scale prospective observational and comparative studies with comprehensive safety assessment protocols are needed to confirm the effectiveness and safety profile of JGT in treating delayed union. While our cases showed no adverse events and previous studies demonstrated no acute toxicity (18), long-term safety data, potential herb-drug interactions, and dose-dependent effects require systematic evaluation in larger populations. Second, some herbs were added to the basic JGT prescription based on each patient’s condition. This reflects the real-world clinical practice in South Korea, where most herbal medicines prescribed by Korean Medicine doctors are customized individually (24). Future studies should focus on identifying the optimal timing and duration of JGT administration as well as investigating potential synergistic effects when combined with conventional treatments. Further research is required to elucidate the mechanisms of action of JGT, particularly regarding its key bioactive compounds.

The present study provides clinical and scientific evidence for the effectiveness of JGT in fracture healing. These findings suggest that JGT could be a valuable complementary addition to the current treatment options for delayed union, particularly as an adjunctive therapy in cases where conventional treatments alone have been insufficient or when surgical intervention is not feasible.

## 4 Conclusion

This case report indicates the effectiveness of JGT for delayed union persisting for more than five months. The therapeutic effects of JGT, supported by radiological evidence and patient-reported outcomes, may be attributed to its bioactive compounds that promote bone regeneration through multiple mechanisms, including enhanced vascular function and osteoblast differentiation. These findings suggest that JGT could be considered as a complementary treatment option for delayed union, particularly when conventional treatments have yielded limited success. However, larger clinical studies are warranted to validate these results.

## Data Availability

The raw data supporting the conclusions of this article will be made available by the authors, without undue reservation.
