# Supplementary material for: Case Report: Therapeutic potential of traditional Korean herbal medicine Jeopgol-tang in bone regeneration: a case series of delayed union over 5 months
Source: Front Endocrinol (Lausanne). 2025 Jun 30;16:1595784. doi: 10.3389/fendo.2025.1595784 (PMC12256232; doi:10.3389/fendo.2025.1595784)
Supplement: Supplementary file 1 [file DataSheet1.pdf]

CARE case report guidelines

CARE Checklist of information to include when writing a case report

(cc) BY-NC-ND

Topic	Item	Checklist item description	Reported on Line
Title	1	The diagnosis or intervention of primary focus followed by the words "case report"	1-3
Key Words	2	2 to 5 key words that identify diagnoses or interventions in this case report, including "case report"	18
Abstract (no references)	3a	Introduction: What is unique about this case and what does it add to the scientific literature?	20-24
	3b	Main symptoms and/or important clinical findings	24-26
	3c	The main diagnoses, therapeutic interventions, and outcomes	26-29
	3d	Conclusion—What is the main "take-away" lesson(s) from this case?	29-32
Introduction	4	One or two paragraphs summarizing why this case is unique (**may include references**)	34-67
Patient Information	5a	De-identified patient specific information.	71-72, 83-84, 94-95
	5b	Primary concerns and symptoms of the patient.	71-75, 83-89, 94-99
	5c	Medical, family, and psycho-social history including relevant genetic information	72, 116-117
	5d	Relevant past interventions with outcomes	73-75, 84-87, 95-99
Clinical Findings	6	Describe significant physical examination (PE) and important clinical findings.	108-109
Timeline	7	Historical and current information from this episode of care organized as a timeline	Figure 4
Diagnostic Assessment	8a	Diagnostic testing (such as PE, laboratory testing, imaging, surveys).	108-109
	8b	Diagnostic challenges (such as access to testing, financial, or cultural)	109
	8c	Diagnosis (including other diagnoses considered)	71-72, 83-84, 94-95
	8d	Prognosis (such as staging in oncology) where applicable	Not applicable
Therapeutic Intervention	9a	Types of therapeutic intervention (such as pharmacologic, surgical, preventive, self-care)	111-112
	9b	Administration of therapeutic intervention (such as dosage, strength, duration)	Table 1, 111-112
	9c	Changes in therapeutic intervention (with rationale)	112-118
Follow-up and Outcomes	10a	Clinician and patient-assessed outcomes (if available)	77-81, 90-92, 101-106
	10b	Important follow-up diagnostic and other test results	Figure 1-3
	10c	Intervention adherence and tolerability (How was this assessed?)	118-120
	10d	Adverse and unanticipated events	120-121
Discussion	11a	A scientific discussion of the strengths AND limitations associated with this case report	150-158, 159-167, 168-169
	11b	Discussion of the relevant medical literature **with references**.	139-149
	11c	The scientific rationale for any conclusions (including assessment of possible causes)	150-158
	11d	The primary "take-away" lessons of this case report (without references) in a one paragraph conclusion	172-178
Patient Perspective	12	The patient should share their perspective in one to two paragraphs on the treatment(s) they received	124-132
Informed Consent	13	Did the patient give informed consent? Please provide if requested	Yes ☒ No ☐
