# Supplementary material for: Case Report: Therapeutic potential of traditional Korean herbal medicine Jeopgol-tang in bone regeneration: a case series of delayed union over 5 months
Source: Front Endocrinol (Lausanne). 2025 Jun 30;16:1595784. doi: 10.3389/fendo.2025.1595784 (PMC12256232; doi:10.3389/fendo.2025.1595784)
Supplement: Supplementary file 2 [file Table1.docx]

**Supplementary table 1.** Composition of Jeopgol-tang.

| Herbal name | Case 1 | Case 2 | Case 3 | | | |
| --- | --- | --- | --- | --- | --- | --- |
|  |  |  | Period 1 | Period 2 | Period 3 | Period 4 |
| Angelicae Gigants Radix | 20g | 20g | 20g | 20g | 20g | 20g |
| Dendrobii Caulis | 16g | 16g | 12g | 12g | 12g | 16g |
| Dipsaci Radix | 12g | 12g | 4g | 4g | 4g | 12g |
| Cnidii Rhizoma | 8g | 8g | 20g | 20g | 20g | 8g |
| Astragali Radix | 8g | 8g | 4g | 4g | 4g | 8g |
| Citri Unshius Pericarpium | 4g | 4g | 8g | 16g | 16g | 8g |
| Cervi Cornu | 10g | 10g | 10g | 10g | 10g | 10g |
| Achyranthis Radix | 4g | 4g |  |  |  | 4g |
| Psoraleae Semen |  | 4g | 8g | 8g | 8g |  |
| Cuscutae Semen |  | 4g | 4g | 4g | 4g |  |
| Massa Medicata Fermentata |  | 4g |  |  |  |  |
| Zingiberis Rhizoma |  | 2.86g |  |  |  |  |
| Zizyphi Fructus |  | 2.86g |  |  |  |  |
| Lycii Fructus |  |  | 10g | 10g | 10g |  |
| Atractylodis Rhizoma Alba |  |  | 4g | 4g | 4g | 4g |
| Ginseng Radix |  |  | 2g | 2g | 2g |  |
| Codonopsis Pilosulae Radix |  |  | 2g | 2g | 2g |  |
| Perillae Folium |  |  |  |  | 4g |  |
| Coptidis Rhizoma |  |  |  |  | 2g |  |
| Cervi Parvum Cornu |  |  | 1g | 1g |  |  |

For Periods 1–4 in Case 3, each herbal prescription was administered for 4 weeks. The prescription dates were as follows: Period 1 to October 12, 2020. Period 2: November 17, 2020–December 14, 2020. Period 3: 9 January 2021; 20 February 2021; 24 March 2021; 1 May 2021. Period 4: December 3, 2021.
